# Charting the trajectory of forgetting: Insights from a working memory period paradigm

**DOI:** 10.3758/s13421-019-00916-6

**Published:** 2019-02-27

**Authors:** John N. Towse, Graham J. Hitch, Neil Horton

**Affiliations:** 1 Lancaster University, Lancaster, UK; 2 University of York, York, UK

**Keywords:** Working memory, Operation period, Individual differences, Forgetting rate

## Abstract

Working memory capacity is commonly measured in terms of its item span, and much less often in terms of its time span, or “period.” The former measures how many items can be stored in working memory when carrying out episodes of concurrent processing. The latter complements this by determining the duration of processing episodes that can be tolerated while successfully storing a fixed number of items. We investigated the generality of previous evidence that working memory period varies with the distribution of longer and shorter processing episodes within a trial, and that notwithstanding such differences, a global measure of period is a reliable predictor of children’s educational attainment. We describe data from 184 children, between 7 and 11 years of age, who completed variants of an operation period task with different distributions of processing episodes together with measures of scholastic attainment. Individual differences in period scores were consistent over two test sessions, and were predictive of reading and number skills. We replicated previous effects of the order of longer and shorter processing episodes, but found that they did not generalize fully to other manipulations of order. The results point to the contribution of subtle within-trial sequence configurations for working memory. We make the case for a broader view of what constrains working memory than exists in current models.

Our goal in this study is to investigate working memory period, a (complementary) alternative to the widespread use of working memory span as a measure of capacity (Towse, Hitch, Hamilton, Peacock, & Hutton, 2005). We introduce the rationale for measuring working memory in this way and examine its characteristics by replicating and then extending previous analyses of both experimental task manipulations and individual differences. Cronbach (1957) emphasized the value of such an integrated approach to experimental and differential approaches 60 years ago, and its relevance for working memory theory has been echoed since (Conway, Jarrold, Kane, Miyake, & Towse, 2007).

The starting point for the present project is the widely held view that working memory is a limited capacity system supporting the maintenance and processing of transient representations (Baddeley, 1986; Baddeley & Hitch, 1974). Within cognitive psychology, the concept of working memory is used to help understand a wide range of phenomena, ranging from the inhibition of saccadic eye movements to such complex activities as playing chess (Crawford, Smith, & Berry, 2017; Robbins et al., 1996). Its success has become intertwined with the enduring popularity of a family of tasks designed to measure working memory capacity, such as counting span, reading span, and operation span (Case, Kurland & Goldberg, 1982; Daneman & Carpenter, 1980; Turner & Engle, 1989).

These span tasks deliberately have a common structure. On a typical trial, participants perform a sequence of processing operations (e.g., counting a display of objects, reading a sentence, carrying out a numerical calculation), and each delivers a memorandum (e.g., an array total, the final word in a sentence, the result of a calculation). At the end of the trial, participants attempt to recall the memoranda in the order they were generated. Trials differ in sequence length, and span estimates the limit on the simultaneous retention of memoranda in working memory. These tasks are referred to generically as complex span tasks, and they assess how much material can be successfully maintained in working memory when attention is also required for processing operations. They predict an impressive range of cognitive and real-world behaviors (see Conway et al., 2007).

Complex span provides tremendous value as a psychometric instrument and has also been deployed in many experiments testing detailed models of working memory processes (e.g., Saito & Miyake, 2004; Towse & Hitch, 1995). Whilst acknowledging this success, one of the potential structural limitations of complex-span tasks is that, inherently, they only measure the number of items that can be recalled when working memory is also engaged in episodes of processing. That is, the paradigm is designed to assess the *residual memory* capacity of the working memory system. As Towse, Hitch, and Horton (2007) note, complex span maps onto a *suitcase* metaphor for the limit on working memory, such that the key variable is how much material can be packed at any one time. Indeed, because complex span is frequently the only task and performance metric, it carries a heavy burden to account for the full range of working memory phenomena researchers may wish to explore (see also Cowan, Morey, Chen, Gilchrist, & Saults, 2008).

Potential alternatives exist to exclusively focus on evaluating span size. Towse et al. (2005) proposed a measure called “working memory period,” designed to assess the endurance or longevity of representations when processing also engages working memory. Underlying this paradigm are two notions: first, that some individuals may be able to withstand a longer filled retention interval than others (i.e., to recall information over a longer period), and second, the possibility that endurance reflects a separable dimension from storage capacity. This notion maps onto an alternative *vacuum flask* metaphor for the limit on working memory (Towse et al., 2007), such that a key variable is how long a flask insulates recently activated material from loss to the ambient environment, over and above its volume.

Towse et al. (2005) reported working memory period data from children. Matching complex span, episodes of processing generate accompanying memoranda. However, unlike complex span, the number of processing episodes remains fixed as trials progress. Instead, the durations of the processing episodes are increased in steps or levels in order to establish the longest overall duration that can be tolerated for successful serial-order recall of the memoranda. For ease of administration and scoring, working memory period is scored in terms of the number of levels through which participants progress. Towse et al. (2005) studied different versions of the period task (a reading period task, analogous to reading span, and operation period, analogous to operation span). They found that working memory period correlated with complex span and with measures of cognitive ability. Furthermore, the period task showed sensitivity to experimental effects that replicated and extended within-trial order effects found previously in span tasks (these are described in more detail below). Thus, whilst initially developed to permit measurement on a separable dimension, working memory period is not entirely orthogonal to working memory span. Towse, Hitch, Hamilton, and Pirrie (2008b) also reported that correct recall times (i.e., production durations) increased with period difficulty level, consistent with the idea that the fidelity of memory representations was degrading and so required more time to be prepared for output.

The importance of rapid forgetting fits with a wide range of developmentally based evidence that faster (general) processing speed is associated with better working memory performance (e.g., the cascade model; Fry & Hale, 1996; Hale, Myerson, Emery, Lawrence, & Dufault, 2007). Potentially, faster processing speed reduces the amount of exposure to information degradation. At a task-specific level, children’s complex span covaries with the speed at which accompanying processing operations can be completed (Bayliss, Jarrold, Gunn, & Baddeley, 2003; Case, Kurland, & Goldberg, 1982; Towse & Hitch, 1995). Moreover, other work converges on the more specific idea that forgetting rate is a separable working memory parameter. In particular, Bayliss and Jarrold (2015) report that one component of working memory capacity variance can be traced to the rate of forgetting in the Peterson and Peterson task (see also the discussion in Jarrold, 2017). Consequently, an endurance-based dimension of working memory, as advocated above, aligns with a range of empirical work. This is broadly consistent with theoretical interpretations—such as the task-switching account (Towse & Hitch, 1995) and the time-based resource-sharing model (TBRS; Barrouillet & Camos, 2007; Barrouillet, Gavens, Vergauwe, Gaillard & Camos, 2009)—that emphasize the importance of rapid forgetting during intervals in which participants undertake processing.

In the present study, we explored the generality of experimental effects in children’s operation period that we had previously interpreted in terms of within-trial forgetting, while seeking further evidence for an association between individual differences in working memory period and scholastic attainment (Towse et al., 2005). We consider these in turn.

## Within-trial forgetting

Towse et al. (2005) found that working memory period showed the same “card order” effects as working memory span (Towse, Hitch, & Hutton, 1998). This terminology reflects the presentation of each processing episode on an image of a card in a computerized display. The logic of card-order effects is as follows. If a trial begins with a short processing episode (card), compared with a long processing episode (card), then the retention demands commence earlier, insofar as retention begins with the generation of the first memorandum (e.g., the result of a math operation or the number of array targets counted). Moreover, if in one condition a trial starts with a short processing episode and ends with a long processing episode, and in another condition this order is reversed, then the total ensemble of processing and storage activities is the same, and any differences in recall are attributable to within-trial order effects.

**Fig. 1:**
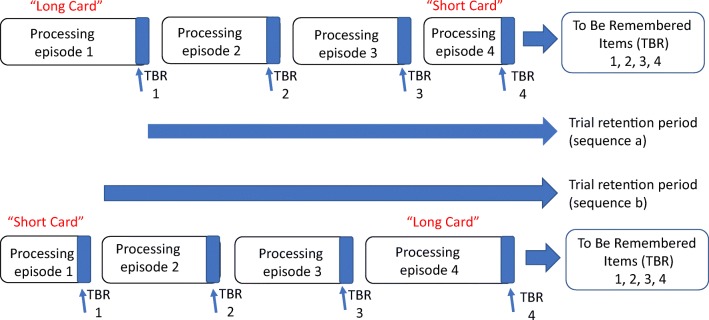
Schematic of four-item working memory span trial, based on Towse et al. (1998). Blue areas for each processing episode represent the identification or specification of the to-be-remembered (TBR) item. The arrows depict the retention phase, which differs between order Sequence a and Sequence b. (Color figure online)

Based on a span paradigm, Towse et al. (1998) reported that “short-final” trials ending with a short processing episode led to higher spans than “long-final” trials did. This occurred for counting span, operation span, and reading span in children (a finding also replicated for counting span by Ransdell & Hecht, 2003), and was taken to support the hypothesis that working memory span is affected by the amount of within-trial forgetting (see also adult data from Maehara & Saito, 2007). Findings were interpreted according to a task-switching model whereby there is no functional opportunity for maintenance during processing episodes which thus serve to postpone the point of recall. This is illustrated in Fig. 1, which shows that a short-final trial (completion Order a) has a shorter overall retention requirement than a long-final trial (completion Order b). The TBRS model of Barrouillet and Camos (2007) differs from the task-switching account because it assumes that “attentional refreshing” during processing episodes can be used to offset forgetting, to an extent that varies inversely with the cognitive load imposed by the processing episodes. However, on this account, too, short-final trials should lead to better performance, benefitting from a combination of a shorter cumulative retention interval and a lower cognitive load.

**Fig. 2:**
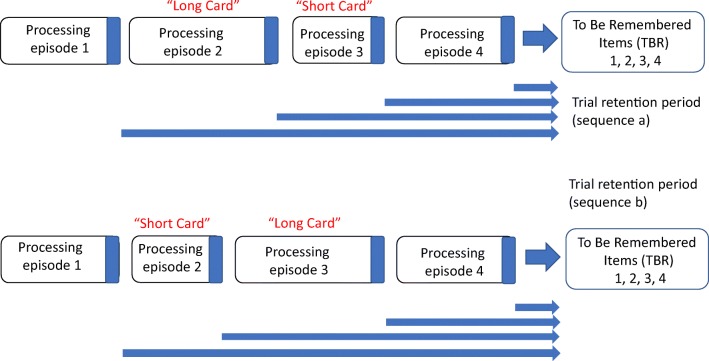
Schematic of a four-item operation period trial. Long and short processing episodes occur at middle positions, which affects (only) the duration of intermediate sequence items. Based on Towse et al. (2005, Fig. 7)

Subsequently, Towse et al. (2005, Experiment 3) showed corresponding order effects in the working memory *period* paradigm, with a working memory advantage for short-final trials of four items than for long-final trials. Towse et al. found a similar working memory advantage when short and long cards were placed in the central rather than at the end positions—period level was larger with a long (second) and short (third) sequence compared with a short (second) and long (third) sequence. Thus, the order effect was not specific to end-item manipulations. This led to an elaboration of the schematic model for retention effects, depicted in Fig. 2. In this case, the durations of processing episodes 2 and 3 were fixed (to be short or long) and those of 1 and 4 were variable and increased progressively through test administration to determine period. A key element to this model is the characterization of a working memory task as the ensemble of a set of item-retention trajectories, not just a unitary composite with a starting and stopping point. Assessing the adequacy of this characterization requires analysis of other novel, sequence permutations. This forms a major element of the present study.

**Fig. 3:**
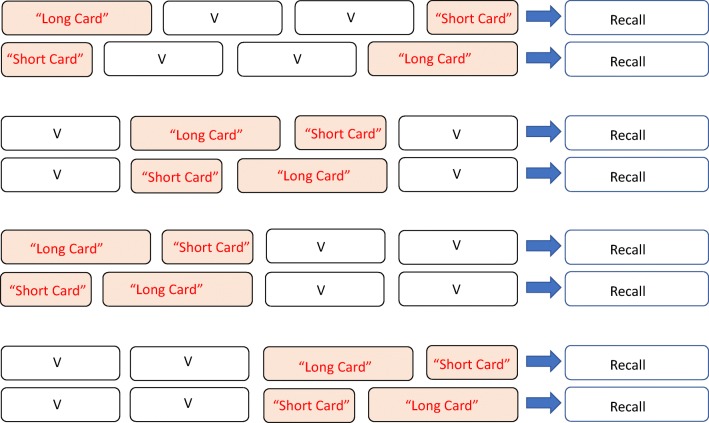
Illustration of the four permutations of the card-order effect manipulated in the study. “V” cards are variables in that they increase with task level. Thus, from top to bottom, this represents LVVS / SVVL; VLSV / VSLV; LSVV / SLVV; VVLS / VVSL. Participants complete each version of the pair in counterbalanced order

In the present study, we sought to replicate and extend these sequence-order manipulations. Therefore, we first created two sequence versions that exactly replicated the order effects described above—manipulating either end positions or middle positions (the outer and inner segments of a four-item sequence). Second, we also created two additional and novel sequences: manipulating order in the first half of a trial and in the second half of a trial. These are all shown in Fig. 3. Together, the sequences explore the conditions under which the ensemble of retention intervals matter for recall accuracy of the set.

It is important to point out that our investigation of these additional conditions was exploratory, in that our main aim was to establish whether previous evidence for the importance of item-retention trajectories in working memory tasks extends to novel permutations of such trajectories. Our task-switching framework is deliberately simplistic, and, consequently, it underspecifies complex patterns of performance. It does, nevertheless, make the general prediction that for each pair of conditions illustrated in Fig. 3, working memory performance will be superior when the short processing episode comes after the long one. Our main objective was to see if we do indeed find such a pattern, as this would encourage further elaboration and testing of more detailed models within the task-switching framework. If, on the other hand, the general prediction is not upheld, this would suggest that the framework is too simplistic, most probably in its assumption that participants are passive throughout. If participants are more active, task strategies represent a powerful extra factor to incorporate within performance.

## Individual differences

A key aspect of complex span is its ability to predict individual differences in higher level cognition. This is applicable to a range of tasks completed by adults (Engle, Kane, & Tuholski, 1999). It is also relevant to children at the primary school level, where complex span predicts aspects of reading and mathematics skill both concurrently and longitudinally (Hitch, Towse, & Hutton, 2001). In this context, an important outcome from previous studies is that working memory period associates with measures of scholastic attainment. Complementing this, working memory period shows some statistical overlap with its better-known span paradigm. This individual-difference perspective supports the idea that the endurance of working memory—or put another way, the rate of forgetting or item loss (Bayliss & Jarrold, 2015)—is a relevant construct that contributes to individual differences and is to some extent distinguishable from capacity measures.

In the present study, we attempted to replicate this link between individual differences in working memory period and scholastic attainment across children of primary school age. In addition, we sought to confirm the reliability of the period measure (Towse et al., 2005, Experiment 1 test–retest reliability estimate was .72). We also investigated whether any relationship between working memory and scholastic attainment could be isolated to particular segments—that is, serial positions—within a working memory period sequence. The fixed list-length architecture of the period paradigm makes this a feasible question to address systematically, unlike orthodox span trials, wherein list length varies across trials and participants. Recent evidence distinguishing secondary memory (early list segments) and primary memory (late list segments) components of free recall lists add weight to this question (Roome et al., 2014; Unsworth & Engle, 2007), especially since Unsworth and Engle (2007) suggest, at least in adult data, that secondary memory provides the cornerstone of complex span performance.

## Method

We report how we determined our sample size, all data exclusions (if any), all manipulations, and all measures in the study (Simmons, Nelson, & Simonsohn, 2012).

### Participants

The analysis sample comprised 184 children from both rural and urban primary schools in North West England (eight additional children did not complete working memory assessments and are not described further). Date of birth was missing for one child, but for the remaining sample, mean age was 9 years, 6 months (range: 7.8–11.9). There were three age groups separated by class assignments. No more than one week after initial assessment, all but 10 of the sample were available to undertake a second working memory assessment. Written parental consent was provided for each participating child.

Based on prior work (i.e., using comparability, not formal power estimates), sample sizes were initially proposed in a grant funding application. These were adjusted through reviewer-suggested design modifications (to collapse conditions), and finalized through availability of children in class for whom parental consent was provided.

### Procedure

Both working memory assessments were administered over a delay of approximately 1 week, with task completion order varied (absences meant three children could contribute only partial data). In the first round of data collection, children also completed the *British Abilities Scale II* (BAS) subtests for word reading and math attainment

An Apple Macintosh PowerBook 5300c controlled experimental tasks, using a RuntimeRevolution software environment, a form of HyperCard stack programming. Administration of operation period followed the procedure reported in Towse et al. (2005). With the use of laminated instruction cards, the experimenter initially explained that arithmetic sums, of the type shown on the cards, would appear on the computer. The child calculated the answer to a problem and reported this verbally. In addition, eight computer-presented sums (comparable to experimental stimuli) were presented without any concurrent memory task as further practice. Feedback appeared after each response to encourage calculation accuracy.

#### Operation period

Each trial comprised four self-paced sums followed by an auditory and visual cue to recall—verbally—the four answers in serial order. In each experimental condition, two of the processing episodes were fixed, and two were variable (see Fig. 3). For each condition, testing involved sets of three trials at a given stage level, with two variable processing episodes determined by independent data on average solution times (see below). Stage level and thus processing duration progressed in successive sets of three trials. Provided that at least two of the three trials at a given level were recalled correctly, a further set was presented at the next level.^1^ Testing continued in this way until children either failed to recall correctly two of the three trials at a particular level, or they reached the maximum level. An audiovisual message congratulated the child whenever he or she successfully recalled at least two of three lists in a set.

We implemented exactly the task rule as in Towse et al. (2005)—that is, item recall was scored as correct when it matched the derived answer supplied by the child to the original problem, even if their arithmetic calculation was erroneous. When children made multiple processing errors on a trial, they received feedback encouraging processing accuracy.

The corpus of arithmetic problems is described in Hamilton, Towse, Hitch, and Hutton (2001), who used empirical data from an independent sample of children to derive estimates of computational duration and accuracy. Problems were selected for each of seven levels of duration and are depictured in Towse et al. (2005, Fig. 1).

Examples of problems for each duration from Level 1 (short and fast) to Level 7 (long and slow) are as follows: 4 + 0 =; 3 + 1 =; 5 − 1 + 0; 4 + 1 − 1; 3 – 1 + 2; 5 – 1 – 1 + 1; 2 + 1 + 2 – 0 − 1. As can be seen, levels differed with respect to the number of arithmetic steps and the size of computational operations (0, 1, or 2), while keeping the answer the same. Towse et al. (2005) showed that, on average, children took almost twice as long to complete long cards (*M* = 6.1 s, drawn from Stage Level 6, above) than short cards (*M* = 3.2 s, drawn from Stage Level 2).

#### Scholastic attainment

Children received the Number Skills and Word Reading subscales of the BAS, the former in a group setting, the latter individually. Older children began at a higher basal level as per BAS instructions. Both are graded tests of attainment in core curriculum activities. The Number Skills test covers, for example, oral number transcription, written arithmetic computations, and, at higher levels, fractions and long division. Word Reading involves reading aloud a series of (regular and irregular) single words.

## Results

Raw data for this study are available at 10.17635/lancaster/researchdata/260.

Towse et al. (2005) presented operation period data in terms of the highest stage level at which recall was accurate (analogous to the largest list length that permits accurate recall in span tasks). That is, from Levels 1–7, at what point did children fail to recall correctly all the four derived solutions within a trial, for the majority of trials? We report the same measure here, likewise incorporating partial credit for recall accuracy at the terminal trial level (Towse & Hitch, 1995).^2^

### Trial configuration effects

Initially, we segregated the different conditions whereby processing length is varied (i.e., the *card-order effect*) into the four permutations shown in Fig. 3. Towse et al. (2005) reported a working memory advantage in the short-late compared with long-late condition (where short-late reduces the retention profile). We aggregated data over the two test sessions, and we similarly calculated a card-order effect for each participant—that is, the difference between working memory period levels (the period score when short cards appears after long, minus the period score when long cards appeared after short).

**Fig. 4:**
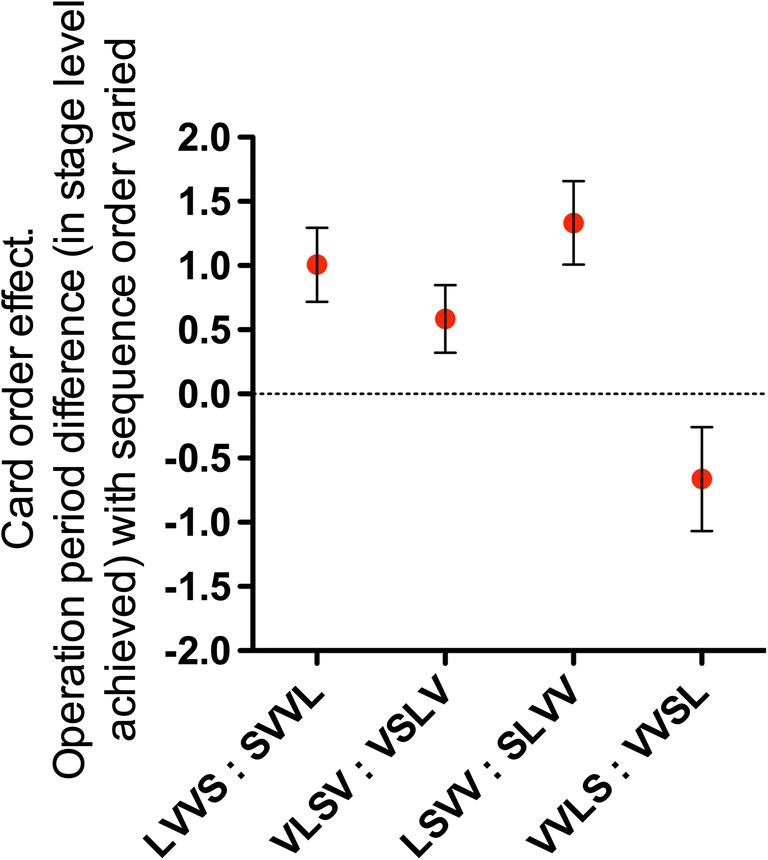
The size and direction of the card-order effect. Operation period difference score in stage level, between a short card following a long card, and a long card following a short card, for each sequence manipulation. Error bars describe one standard error on each side

In the current data, the card-order effect was systematic, positive, and substantial with a short-final (and thus long-first) sequence (i.e., LVVS vs. SVVL in Fig. 3), *t*(43) = 3.492, *p* = .001, η^2^ = .221. This is illustrated in Fig. 4. Next, we examined the card-order effect established by Towse et al. (2005), where short and long late cards are manipulated in central rather than end positions, (i.e., VLSV vs. VSLV in Fig. 3). We again found a positive and systematic short-late recall advantage, *t*(43) = 2.213, *p* = .032, η^2^ = .102.

We then tested what is, to our knowledge, a novel order effect, where short and long cards are both placed in the first half of the sequence (i.e., LSVV vs. SLVV). As with the configurations assessed above, this also yielded a systematic short-late advantage, *t*(42) = 4.098, *p* < .001, η^2^ = .286. The effect size was again substantial. Of course, the term *short-late* here is relative, since both short and long cards are positioned in the first half of the sequence.

Finally, we examined the effect of manipulating the order effect in the second half of the sequence, which forms the natural complement to the previous configuration (i.e., VVLS vs. VVSL). In this instance, there was no systematic short-late advantage, *t*(42) = −1.632, *p* = .110, η^2^ = .060, and the effect size was the smallest of the comparisons made. The contrast between this configuration and others is also evident in Fig. 4.

These analyses provide a focused investigation of the card-order effect in its various permutations, comparable with Towse et al. (2005). However, we observed that task performance in general shows a robust and sizeable practice or repetition effect, with a 30.2% improvement in operation period on the second administration, *t*(173) = 5.038, *p* < .001, η^2^ = .128. The above card order analyses collapse across this general improvement in performance, over which card-order configurations were counterbalanced. Therefore, we next examined the relationship between card-order effects and session.

**Fig. 5:**
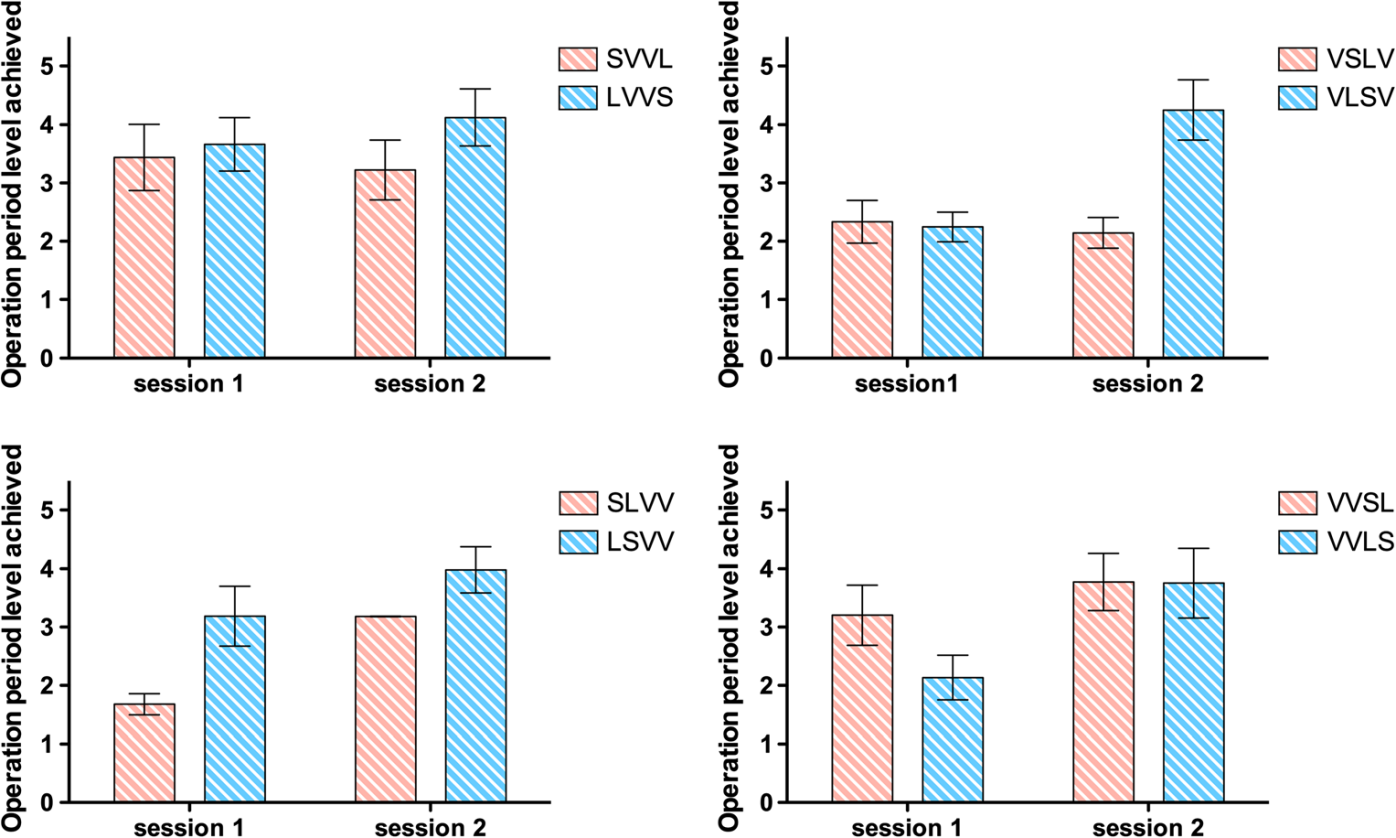
Card-order effects and session effects for period level achieved. Error bars describe one standard error on each side

First, we examined the original card order effect (separating SVVL followed by LVVS from LVVS followed by SVVL), finding a significant interaction with session, *F*(1, 42) = 4.603, *p* = .038, η_p_^2^ = .099. As can be seen in Fig. 5, the short-final advantage was stronger in the second session. This prompted us to reanalyze data originally reported by Towse et al. (2005). A (previously unanalyzed) session by card-order effect interaction was evident there also. Thus, the pattern reported here is not unique to the current study.

Second, we examined the card-order effect in central rather than terminal positions (separating VSLV followed by VLSV from VLSV followed by VLSV). This also yielded a significant interaction with session, *F*(1, 42) = 14.938, *p* < .001, η_p_^2^ = .262. Again, the short-late advantage was stronger when tested in the second session.^3^

Third, we examined short and long cards manipulated in the first half of the sequence (SLVV and LSVV alongside its counterbalanced pair). We found once more an interaction with session, *F*(1, 41) = 18.078, *p* < .001, η_p_^2^ = .306. In this case, the recall advantage to a short-late card was stronger in the first session comparison.^4^

Finally, we examined the effect of manipulating the card-order effect in the second half of the sequence (VVSL and VVLS, alongside the counterbalanced match). In this case, we did not obtain a reliable interaction with session, *F*(1, 41) = 2.427, *p* = .127, η_p_^2^ = .056. The main effect (i.e., benefit) of practice was reliable, *F*(1, 41) = 7.454, *p* = .009, η_p_^2^ = .154, while the difference between orders was not (*F* < 1, η_p_^2^ = .020).

We consider the implications of these card-order effects, and the asymmetric transfer effects across session, in the Discussion. At this point, we simply note that working memory endurance is systematically affected by several, but not all, sequence permutations, with large effect sizes in two cases, a moderate effect in one, and a small-moderate reverse effect in the condition where order permutation did not significantly affect recall. Thus, previous card-order phenomena have been replicated and extended, but interestingly, a short-late advantage is not found under all circumstances.

**Fig. 6:**
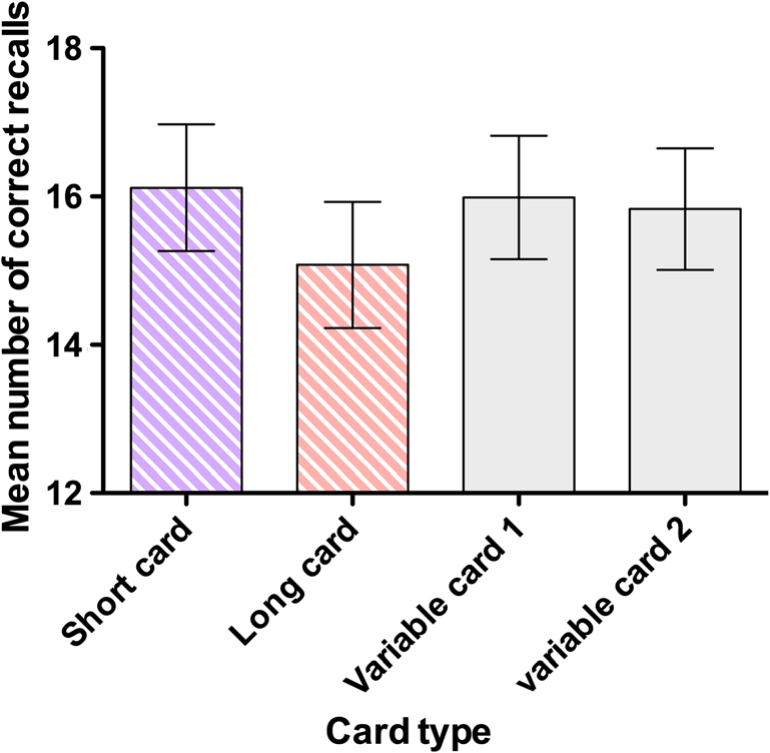
Recall performance associated with the card type products. Error bars describe one standard error on each side

The data also permit a different type of question to be investigated focusing on recall accuracy associated with each type of processing card (i.e., long, short, first variable card, second variable card). This is described in Fig. 6. Analysis confirmed that answers to long cards were less well recalled than were answers to short cards *t*(183) = 6.993, *p* < .001, η^2^ = .211, and variable cards (e.g., long vs. first variable card), *t*(183) = 6.579, *p* < .001, η^2^ = .211, while the two variable cards were not significantly distinguishable, *t*(183) = 1.328, *p* = .186, η^2^ = .010. We defer interpretation of these data to the Discussion.

### Arithmetic operations: Processing time analysis

**Fig. 7:**
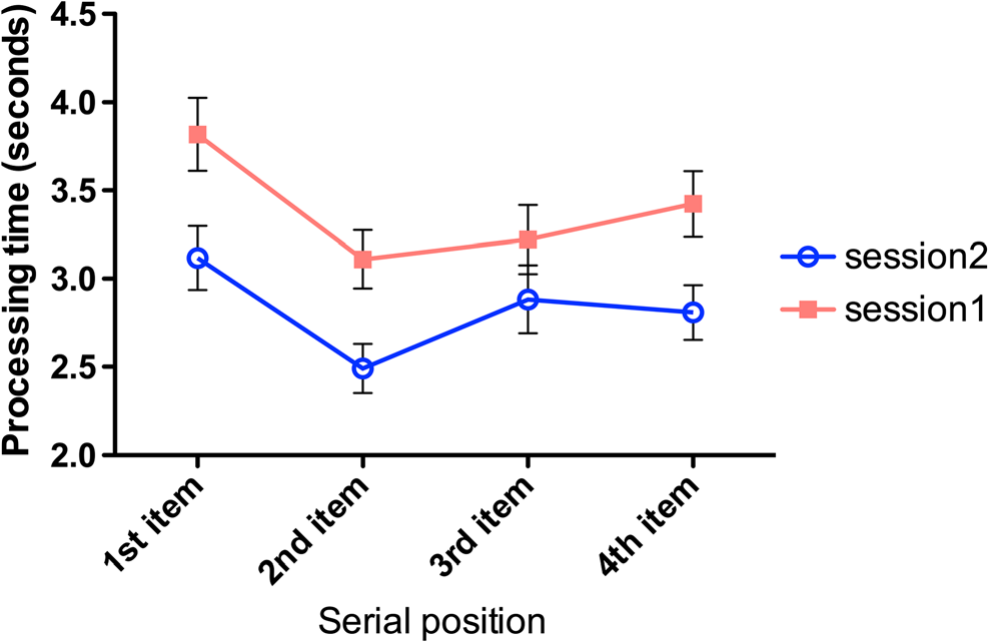
Processing times for arithmetic problems as a function of serial position and session. Error bars describe one standard error on each side

To analyze the time taken to respond to arithmetic problems, we based performance on the first set of three trials only (and not subsequent sets, even when these were potentially available). Even though this produced a sparse data set, we wanted to derive a measure that was equivalent (all children experienced the first set of trials) and thus comparable (beyond the first set of trials, arithmetic operations changed, so the sampling space differed). Overall performance is described in Fig. 7. These data emphasize several features. First, solution times on the second session are consistently quicker than those on the first session (approximately .5 s for each answer calculated, or 2 s from the initial problem presentation time until the recall cue). This offers one insight into the practice benefits in the memory recall data—on the second session, quicker responses reduced the retention duration for the trial by more than 10%.

Second, for neither session do the data exhibit a monotonic increase in duration across serial position. This is noteworthy insofar as accounts of working memory that draw upon the notion of resource sharing or a trade-off between processing and memory (Barrouillet et al., 2004; Case et al., 1982) should predict that processing times will be longer at later serial positions because of the increasing burden of handling computations alongside retention of prior answers. Furthermore, even if one accepts that the first serial position may be affected by “start of trial” or “startup” costs. we found no main effect of serial position when examining just serial positions 2–4, *F*(2, 182) = 1.896, *p* = .153, η_p_^2^ = .020; there was a strong session effect, *F*(1, 183) = 22.9, *p* < .001, η_p_^2^ = .109, but no reliable interaction (*F* < 1, *p* = .650, η_p_^2^ = .005).

### Recall accuracy as a function of serial position

**Fig. 8:**
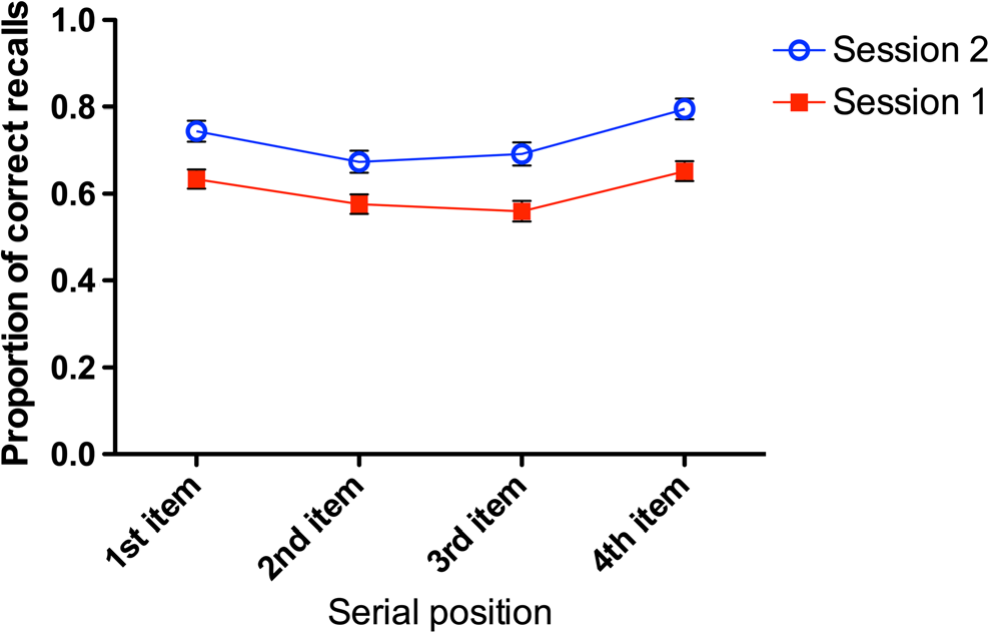
Proportion of correct recalls as a function of serial position and session. Error bars describe one standard error on each side

We calculated the accuracy of recall as a function of each serial position on the first three task trials (i.e., Level 1). These are reported in Fig. 8 for the two sessions. A conventional (albeit mild), bow-shaped recall function replicates the pattern of data reported in Towse et al. (2005; see Fig. 2). The data also illustrate the improved second session recall that is visible at each serial position. More detailed analysis of serial position accuracy across period task levels is reported in the Appendix.

### Individual differences

We have already noted that in absolute terms operation period performance improved from Session 1 to Session 2. However, performance also showed systematic stability with respect to individual differences. Working memory period correlated across sessions, *r*(172) =.514, *p* < .001, and processing speed—which is itself a common associate/mediator of children’s working memory performance—was also consistent across sessions, *r*(172) = .619, *p* < .001. The reliability estimate of period is strong, although slightly smaller than reported in Towse et al. (2005), which was derived from a smaller and more restricted age sample, without the different card-order permutations and a slightly different scoring algorithm (based on aggregating different recall accuracy thresholds). Table 1 reports the relationships between the principal variables of interest in the study. Six children had missing data on the BAS Number Skills score, four children had missing data on Word Reading, and two children lacked data on both assessments. We derived a composite measure of number and reading skills by averaging *z* scores on each raw score variable (using just the single *z* score for the partial data noted above). We also created a composite measure of operation period by combining *z* scores from each assessment where available and a measure of arithmetic (operation) processing speed based on solution times for the first set of trials.

**Table 1:** Relationships between variables

	1	2	3	4	5
1. Age (months)					
2. BAS (number)	.597				
3. BAS (word read)	.458	.638			
4. BAS (composite)	.581	.908	.909		
5. Operation period (composite)	.368	.525	.496	.544	.438
6. Processing speed	−.405	−.517	−.470	−.530	−.438

*Note.* BAS = *British Abilities Scale (II)*

Of particular note, working memory period robustly correlated with a composite ability measure from BAS tests, and this relationship remained significant once partialing out children’s age. It was also the case that period was correlated with ability specifically for the youngest age group, *r*(59) = .316, *p* = .013, for the middle age group, *r*(60) = .537, p < .001, and for the oldest age group, *r*(57) = .449, *p* < .001. Moreover, these relationships also persisted after controlling for age (in months) within each age band, with *r*(57) = .316, *p* = .015, *r*(59) = .498, *p*< .001, and *r*(56) = .456, *p* < .001, respectively. The data support the conclusion that representational endurance, as measured by working memory period, is a stable and meaningful characteristic across the sampled age groups, and specifically at each age band, with a numerical trend towards a stronger relationship amongst older children.

For participants with data on all three measures, operation period correlated specifically with both Number Skills, *r*(170) = .489, *p* < .001, and Word Reading, *r*(170) = .449, *p* < .001. These two correlations did not significantly differ in strength (*z* = .71, *p* = .239). That the numerical trend is for a stronger link with number skills is not at all surprising, given that operation period involves simple numerical calculations. Indeed, that these are statistically equivalent reinforces the view that the primary task demand in this configuration of operation period is the retention element and not the processing content.

To summarize, there is a healthy association between the overall operation period score and the aggregated measure of scholastic skills. Since the operation period task uses a fixed list-length structure, it is also feasible to investigate whether this relationship is carried by all elements of the sequence, or whether, for example, early or late serial positions (presumably affected by primacy and recency processes, respectively) differentially contribute to the predictive profile. We calculated participant recall accuracy for each serial position, separately for Sessions 1 and 2, and found a strong convergence in that there were consistent associations between position-specific accuracy and BAS scores, *r*s(182) >.343, all *p*s<.001, for Positions 1–4. Individual differences in ability scores and working memory endurance were not mediated by specific portions of the recall curve, nor were they reliant solely on the aggregation of positional data.

As noted above, we also replicate a finding consistently obtained across multiple studies of children (see Towse & Hitch, 1995)—individual differences in working memory ability are linked the speed with which the processing elements of the task can be accomplished.^5^ Children who quickly answer the arithmetic problems tend to be children with larger working memories—here, they are children who can endure a longer retention period and still effectively access the to-be-remembered items.

## Discussion

In first reporting on the working memory period, paradigm, Towse et al. (2005) argued that “the present research represents an important description of the *potential* of a novel measure of working memory, p569.” Our study confirms and extends that potential, with respect to individual differences and experimental analysis. To justify this claim, we discuss the terminology adopted, key findings, and their interpretation.

In this study, card-order effects have been specified by referring to a short-late advantage in sequence order. We should note that this descriptive label is partly one of convenience only, since the data are not available to demonstrate unequivocally whether it is primarily a short-late advantage—foreshortening the delivery of a recall cue—or a long-late disadvantage—delaying the delivery of a recall cue—or, indeed, an amalgam of both. We believe it would require a different type of experimental design to arbitrate satisfactorily between these possibilities.

We introduced the card order analyses with respect to the schematic model in Fig. 1. The key feature captured by that model was that short-final and long-final sequences can be distinguished by when retention commences. This model assumes that recall begins earlier and occupies more time in the long-final (short-first) condition. That model was later elaborated into a second version, Fig. 2 (Towse et al., 2005), by making the explicit distinction between overall recall duration for the trial and trajectories for each item in the set. This elaboration helped to understand the finding of a short-late and not just short-final advantage.

We have replicated both the short-final and short-late advantage with working memory period. This confirms the important but often overlooked conclusion that working memory retention and recall is not a single act. Instead, memoranda can exist at different levels of fidelity. In addition, we have also reported data reinforcing the view that the task-switching model, and its family neighbors, even where it explains the majority of effects, does not explain the totality of data.

In particular, whilst there is evidence that a short-late advantage can be obtained when short and long processing episodes appear in the first half of the ensemble, there is no comparable advantage when they appear in the second half of the ensemble. Also, we found that card-order effects vary with session repetition, for which task switching does not offer a simple explanation. Accordingly, whilst the data confirm the importance of taking detailed, within-trial temporal perspectives into account, modeling temporal trial effects is likely insufficient on its own.

The impetus for the present work has been to explore predictions from a simple task-switching hypothesis (Towse & Hitch, 1995; Towse et al., 2005; Towse et al., 1998), though this account is not alone in proposing temporal constraints on working memory performance. In particular, the TBRS (e.g., Barrouillet & Camos, 2014) includes a loss-and-refresh cycle for representational fidelity based on multiple rehearsals through a trial (micro task switches; see also Towse et al., 2002). For example, Portrat and Lemaire (2015) model successive decay and refresh trajectories of a single item over an interval, where decay weights more strongly than does refresh, and leads overall to loss of activation (see Fig. 1, Portrat & Lemaire, 2015). This is in many ways a nonlinear version of Fig. 1, focusing on a single memorandum.

Consistent with Portrat and Lemaire (2015), the empirical data point to the heuristic value of considering the ensemble activation and time parameters of a working memory trial. Data also emphasize the relevance of how individual representations are retained. Yet none of the current models are complete, because some sequence order manipulations do not impact recall in the predicted way. In this respect, the current patterns of data present challenges for both the task-switching account and the TBRS model, because within the loss-and-refresh perspective in the latter model, longer cards should generate additional decay through attentional processes switched away from all working memory representations at that point.

In summary, most card-order manipulations confirm the importance of item retention, but they defy a simple, unitary account. One possible interpretation from Fig. 4 is that the largest effect occurs when short and long cards are manipulated in the first two positions, and the next largest effect occurs when a short or long card is placed in the first position. Placement of short/long cards in the middle positions generates a smaller effect, and placing them in the final two positions leads to the smallest (negative) effect. In other words, a post hoc description of the data is that larger card-order effects occur when they are placed early within the serial position sequence, and smaller effects occur when they are placed later in the serial position sequence.

How might these inconsistent sequence permutation effects be explained? In short, we do not have a comprehensive answer. However, one potential clue to understanding all the models’ limitations lies in the large main effect of practice on working memory period and interactions whereby card-order effects change with practice across session. These interactions suggest that recall benefits from processing speed changes and from the deployment of different performance strategies. Different refreshment strategies have been modeled in other work (see Lemaire, Pageot, Plancher, & Portrat, 2017), and whilst we cannot identify the specific strategies that account for the practice effects, their presence serves as an important reminder that almost all existing models are dependent on essentially unexplored assumptions about strategies. Strategy changes can be rapid, occurring within a session (Towse et al., 2008a), and they can also be slower and longer-term, underlying working memory training (Guye & von Bastian, 2017; Stone, 2016). In terms of the Baddeley and Hitch (1974) working memory framework, these data highlight the need for modeling the *flexibility* with which central executive processes can be deployed to support working memory.

A second clue comes from the observation that answers from long cards were harder to remember than the answers from short cards, with the latter more closely resembling variable length cards (see Fig. 6). The equivalence between memory for the answers to short and variable cards may have arisen from many children faltering at a fairly early stage of the period task. This is because in the determination of working memory period, variable cards start off more similar to short cards, and then progressively become more like long cards as the level of difficulty is increased. The greater difficulty of remembering answers to long cards is striking evidence that period is influenced by factors other than within-task retention intervals. We note that long cards involved more arithmetic operations and therefore more interim results, and we suggest that these may interfere with memory for the final result.

Irrespective of these specific interpretations, data underline the value of measuring working memory period. That is, the attempt to measure the endurance of working memory representations, whilst keeping constant the number of independent items held in memory, is illuminating. We note that it would be very difficult to measure different permutations of card-order effects within a span paradigm because list length must vary across trials in order to assess capacity. Likewise, it would be hard to determine the selective contribution of recall at specific serial positions for predictive power (as we consider in more detail below) when the serial positions vary with list length. In summary, the characteristics of working memory period afford novel perspectives into some of the processes that support working memory performance.

It is clear that explaining the retention requirements of representations is important—as within-task retention duration increases, the probability of correct recall declines. Yet whilst this is the case for many card-order sequences, it is not true for all of them. The present data implicate a number of other factors that determine the period of working memory, including processing speed and practice effects, strategies, and interference (see also Posner & Konick, 1966). This argues against simple models, but without detracting from the value of measuring working memory period.

The value of working memory period is also demonstrated through the evidence that children’s performance correlates robustly with scholastic attainment—indeed, despite large practice effects—and this is also true throughout the serial position list. Empirically, an index of representational endurance is shown to be both stable and linked to scholastic ability—there is reliability and predictive validity. Importantly, this offers converging evidence that forgetting rate is a relevant attribute of working memory (see also, for example, Jarrold, 2017). Forgetting rate often has been overlooked in studies of complex working memory that focus instead on capacity, but it is increasingly apparent that it affects performance, and there is growing evidence that we can develop tasks to successfully capture this parameter.

Just as we have advocated the value of implementing a period procedure for illuminating working memory issues, we should also point out some of the arguments we are explicitly *not* making. First, as should be clear from what we have said above, we do not wish to claim that time is necessarily the causal *mechanism* for informational loss. For example, the task structure always delivers four TBR items and has easier trials to start with. Being more likely to be correctly remembered, these items are thereby available to interfere with subsequent trials, clearly providing the opportunity for the buildup of proactive interference across trials. To clarify—our proposal is that endurance is a useful metric for measuring working memory, not a simple temporal causal mechanism of forgetting.

Second, whilst the period task keeps list length constant, we do not claim that volume-related or capacity-related issues do not contribute to period task demands. There is a constant volume of things to remember, which is likely nontrivial for some children. These two concepts are intertwined; in just the same way, a working memory capacity metric is not an instantaneous trial format, and thus involves endurance. We refer to different metaphors for memory—suitcases and vacuum flasks (Towse et al., 2007). We regard these as useful perspectives that highlight relevant dimensions. Yet these dimensions cannot be entirely orthogonal and independent, and both neglect the important role of, for example, executive processes in complex task performance. And third, we are not claiming that capacity, endurance, or speed of processing represents the sole constraint on working memory performance. There is abundant evidence for other contributory processes that shape the quality of encoding (e.g., chunking; Cowan, 2010), maintenance (e.g., mapping onto longer-structure representations; Ericsson & Delaney, 1999), and recall (e.g., recall reconstruction; Towse et al., 2010).

In conclusion, we reiterate that it is valuable conceptually to show that working memory phenomena do not completely rely on a single paradigm family focusing on volume metrics. That most theoretical and empirical working memory research is informed by some version of a suitcase measurement metaphor is an important recognition in its own right. The present data show that this can be usefully augmented by modeling the flask-like properties of memory. Highlighting the endurance of memory representation does not offer a sufficient or complete account of working memory, yet it is, we argue feasible, tenable, coherent, and informative.

## Figures and Tables

**Fig. 9:**
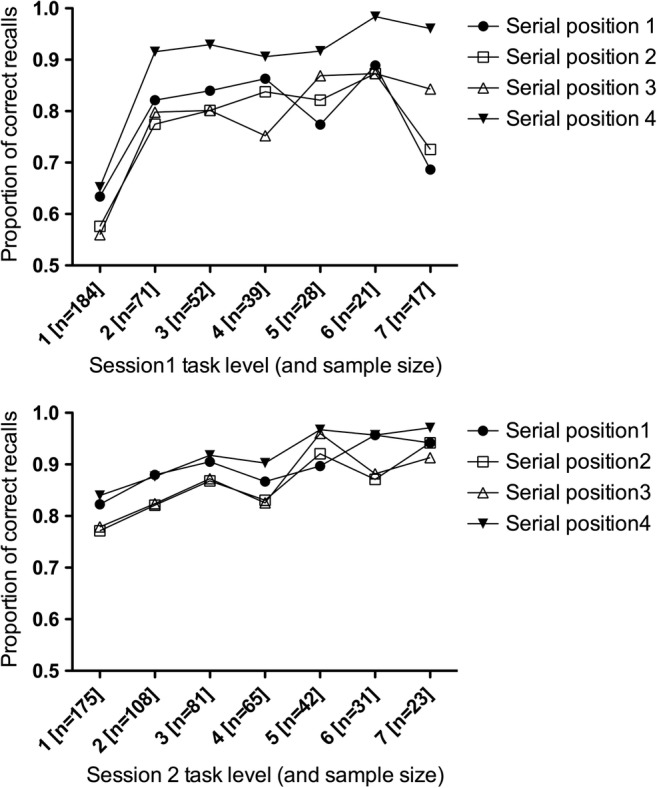
Proportion of correct recalls as a function of task level and serial position. Upper panel represents data from Session 1; lower panel represents data from Session 2

## Appendix

Serial position analysis is reported in the main text. A more detailed analysis is also possible that describes recall in terms of the period test level administered (i.e., the recall difficulty in terms of the endurance required of representations). These data are reported in Fig. 9. It may seem paradoxical in these data that accuracy *increases* with difficulty level. However, later test levels are based on successively smaller samples (these are specified on the *x*-axis labels), and thus comprise performance from the more able children. Figure 9 also demonstrates that the serial position curve is not constant across all task levels—in particular, for Session 1, the primacy advantage evident at the start of performance declines. In comparison, for Session 2 data, the primacy and recency effects are more evident throughout.

## Footnotes

1 An early version of period software was developed and made available here (http://www.lancs.ac.uk/staff/towse/wmperiod.html), and a configurable software implementation of the working memory period paradigm, written by James Stone, also available here (http://www.cognitivetools.uk/cognition/tasks/Verbal-WM/workingMemoryPeriod/).

2 The accompanying raw data also document performance based on the number of successful list recalls (see Conway et al., 2005, for an analysis of analogous working memory span scoring algorithms). Statistical conclusions are not affected by choice of scoring measure.

3 In this analysis, we also found a significant overall practice effect, *F*(1, 42) = 10.686, *p* = .002, η_p_^2^ = .203, and a difference between the two orders, *F*(1, 42) = 5.902, *p* = .019, η_p_^2^ = .123.

4 Here, the main effect (i.e., benefit) of practice was also significant, *F*(1, 41) = 15.086, *p* < .001, η_p_^2^ = .269, but no systematic difference between order configuration (*F* < 1, η_p_^2^ = .015).

5 Processing speed is measured with the first set of trials only, so as to avoid the confound of mixing different trial sets that have different length configurations.

## References

[CR1] Baddeley AD (1986). Working memory.

[CR2] Baddeley AD, Hitch GJ, Bower GH (1974). Working memory. The psychology of learning and motivation: Advances in research and theory.

[CR3] Barrouillet P, Camos V, Osaka N, Logie RH, D’Esposito M (2014). The time-based resource-sharing model of working memory. The cognitive neuroscience of working memory.

[CR4] Barrouillet P, Camos V (2014). Working memory: Loss and reconstruction.

[CR5] Barrouillet P, Gavens N, Vergauwe E, Gaillard V, Camos V (2009). Working memory span development: A time-based resource-sharing model account. Developmental Psychology.

[CR6] Bayliss DM, Jarrold C (2015). How quickly they forget: The relationship between forgetting and working memory performance. Journal of Experimental Psychology: Learning, Memory, and Cognition.

[CR7] Bayliss DM, Jarrold C, Gunn DM, Baddeley AD (2003). The complexities of complex span: Explaining individual differences in working memory in children and adults. Journal of Experimental Psychology: General.

[CR8] Case R, Kurland M, Goldberg J (1982). Operational efficiency and the growth of short term memory span. Journal of Experimental Child Psychology.

[CR9] Cronbach LJ (1957). The two disciplines of scientific psychology. American Psychologist.

[CR10] Conway ARA, Kane MJ, Bunting MF, Hambrick DZ, Wilhelm O, Engle RW (2005). Working memory span tasks: A methodological review and user's guide. Psychonomic Bulletin & Review.

[CR11] Conway AR, Jarrold C, Kane M, Miyake A, Towse JN (2007). Variation in working memory.

[CR12] Cowan N (2010). The magical mystery four: How is working memory capacity limited, and why?. Current Directions in Psychological Science.

[CR13] Cowan N, Morey CC, Chen Z, Gilchrist AL, Saults JS, Ross BH (2008). Theory and measurement of working memory capacity limits. The psychology of learning and motivation.

[CR14] Crawford TJ, Smith ES, Berry DM (2017). Eye gaze and aging: Selective and combined effects of working memory and inhibitory control. Frontiers in Human Neuroscience.

[CR15] Daneman M, Carpenter PA (1980). Individual differences in working memory and reading. Journal of Verbal Learning and Verbal Behavior.

[CR16] Engle RW, Kane MJ, Tuholski SW, Miyake A, Shah P (1999). Individual differences in working memory capacity and what they tell us about controlled attention, general fluid intelligence, and functions of the prefrontal cortex. Models of working memory.

[CR17] Ericsson KA, Delaney PF, Miyake A, Shah P (1999). Long-term working memory as an alternative to capacity models of working memory in everyday skilled performance. Models of working memory.

[CR18] Fry AF, Hale S (1996). Processing speed, working memory, and fluid intelligence. Psychological Science.

[CR19] Guye, S., & von Bastian, C.C. (2017). Working memory training in older adults: Bayesian evidence supporting the absence of transfer. *Psychology and Aging.* doi:10.1037/pag0000206 10.1037/pag0000206 29239658

[CR20] Hale S, Myerson J, Emery LJ, Lawrence BM, Dufault C, Conway ARA, Jarrold C, Kane MJ, Miyake A, Towse JN (2007). Variation in working memory across the life span. Variation in working memory.

[CR21] Hamilton, Z., Towse, J. N., Hitch, G. J., & Hutton, U. (2001). *Analysis of solutions to arithmetic operations differing in the number of computational terms* (Technical Report, CDRG8). Retrieved from http://www.lancaster.ac.uk/staff/towse/papers/cdrg8.pdf

[CR22] Hitch GJ, Towse JN, Hutton UMZ (2001). What limits working memory span? Theoretical accounts and applications for scholastic development. Journal of Experimental Psychology: General.

[CR23] Jarrold C (2017). Working out how working memory works: Evidence from typical and atypical development. Quarterly Journal of Experimental Psychology.

[CR24] Lemaire, B., Pageot, A., Plancher, G., & Portrat, S. (2017). What is the time course of working memory attentional refreshing? *Psychonomic Bulletin & Review*. doi:10.3758/s13423-017-1282-z 10.3758/s13423-017-1282-z 28364345

[CR25] Maehara Y, Saito S (2007). The relationship between processing and storage in working memory span: Not two sides of the same coin. Journal of Memory and Language.

[CR26] Posner MI, Konick AF (1966). On the role of interference in short-term retention. Journal of Experimental Psychology.

[CR27] Portrat S, Lemaire B (2015). Is attentional refreshing in working memory sequential? A computational modeling approach. Cognitive Computation.

[CR28] Ransdell S, Hecht SA (2003). Time and resource limits on working memory: Cross age consistency in counting span performance. Journal of Experimental Child Psychology.

[CR29] Robbins, T. W., Anderson, E. J., Barker, D. R., Bradley, A. C., Fearneyhough, C., Henson, R. N. A., . . . Baddeley, A. D. (1996). Working memory in chess. *Memory and Cognition, 24*, 83–93. 10.3758/bf03197274 8822160

[CR30] Roome, H., Towse, J., & Jarrold, C. (2014). How do selective attentional processes contribute to maintenance and recall in children’s working memory capacity? *Frontiers in Human Neuroscience, 8*(1011). doi:10.3389/fnhum.2014.01011 10.3389/fnhum.2014.01011 PMC4267211 25566031

[CR31] Saito S, Miyake A (2004). On the nature of forgetting and the processing-storage relationship in reading span performance. Journal of Memory and Language.

[CR32] Simmons, J. P., Nelson, L. D., Simonsohn, U. (2012). *A 21 word solution* Retrieved from ssrn.com/abstract=2160588

[CR33] Stone, J. (2016). *How does practice affect working memory? The efficacy of adaptive-difficulty working memory training programs* (Doctoral thesis, Lancaster University, Lancashire, UK). Retrieved from http://eprints.lancs.ac.uk/85682/

[CR34] Towse JN, Cowan N, Horton N, Whytock S (2008). Task experience and children’s working memory performance: A perspective from recall timing. Developmental Psychology.

[CR35] Towse JN, Hitch GJ (1995). Is there a relationship between task demand and storage space in tests of working memory capacity?. Quarterly Journal of Experimental Psychology.

[CR36] Towse, J. N., Hitch, G. J., & Hutton, U. (2002). On the nature of the relationship between processing activity and item retention in children. *Journal of Experimental Child Psychology, 82*(2), 156–184. 10.1016/s0022-0965(02)00003-6 12083794

[CR37] Towse JN, Hitch GJ, Hamilton Z, Peacock K, Hutton UMZ (2005). Working memory period: The endurance of mental representations. Quarterly Journal of Experimental Psychology.

[CR38] Towse JN, Hitch GJ, Hamilton Z, Pirrie S (2008). The endurance of children’s working memory: A recall time analysis. Journal of Experimental Child Psychology.

[CR39] Towse JN, Hitch GJ, Horton N (2007). Working memory as the interface between processing and retention: A developmental perspective. Advances in Child Development and Behavior.

[CR40] Towse JN, Hitch GJ, Horton N, Harvey K (2010). Synergies between processing and memory in children’s reading span. Developmental Science.

[CR41] Towse JN, Hitch GJ, Hutton U (1998). A reevaluation of working memory capacity in children. Journal of Memory and Language.

[CR42] Turner ML, Engle RW (1989). Is working memory capacity task dependent?. Journal of Memory and Language.

[CR43] Unsworth N, Engle RW (2007). The nature of individual differences in working memory capacity: Active maintenance in primary memory and controlled search from secondary memory. Psychological Review.

